# Untargeted Metabolomics Analysis of Eggplant (*Solanum melongena* L.) Fruit and Its Correlation to Fruit Morphologies

**DOI:** 10.3390/metabo8030049

**Published:** 2018-09-01

**Authors:** Abu Hanifah, Awang Maharijaya, Sastia P. Putri, Walter A. Laviña, Sobir

**Affiliations:** 1 Department of Agronomy and Horticulture, Bogor Agricultural University, Jl. Meranti, IPB Dramaga Campus Bogor, West Java 16680, Indonesia; abuhanifah.jbr@gmail.com (A.H.); awang.maharijaya@gmail.com (A.M.); 2 Center for Tropical Horticulture Studies, Bogor Agricultural University, Bogor 16680, Indonesia; 3 Department of Biotechnology, Graduate School of Engineering, Osaka University, 2-1 Yamadaoka, Suita, Osaka 565-0871, Japan; sastia_putri@bio.eng.osaka-u.ac.jp; 4 Microbiology Division, Institute of Biological Sciences, University of the Philippines Los Baños, Los Baños, Laguna 4031, Philippines; walavina@up.edu.ph

**Keywords:** untargeted metabolomics, eggplant fruit diversity, GC-MS, LC-MS, eggplant fruit morphology

## Abstract

Eggplant is one of the most widely cultivated vegetables in the world and has high biodiversity in terms of fruit shape, size, and color. Therefore, fruit morphology and nutrient content become important considerations for both consumers and breeders who develop new eggplant-based products. To gain insight on the diversity of eggplant metabolites, twenty-one eggplant accessions were analyzed by untargeted metabolomics using GC-MS and LC-MS. The dataset of eggplant fruit morphologies, and metabolites specific to different eggplant fruit accessions were used for correlation analysis. Untargeted metabolomics analysis using LC-MS and GC-MS was able to detect 136 and 207 peaks, respectively. Fifty-one (51) metabolites from the LC-MS analysis and 207 metabolites from the GC-MS analysis were putatively identified, which included alkaloids, terpenes, terpenoids, fatty acids, and flavonoids. Spearman correlation analysis revealed that 14 fruit morphologies were correlated with several metabolites. This information will be very useful for the development of strategies for eggplant breeding.

## 1. Introduction

Eggplant (*Solanum melongena* L.) is one of the most widely grown vegetables in the world [1,2], ranking sixth in global vegetable production. Eggplants are mainly grown for their fruits, with only a few cases reporting the use of eggplant leaves as herb or for medicinal uses [1]. China has the highest eggplant production in the world with 22 million tons, ca. 62% of the total world production [3]. Eggplant is one of the most common ingredients in Indonesian cuisine and Indonesian people consume local eggplants of different shapes, colors, and patterns. Thus, in this region, different fruit morphology characteristics are considered major factors when assessing the quality of eggplants. These include several morphological characteristics such as color, shape, size, uniformity, shelf-life, and nutrient content.

Nutrient content is an important consideration when choosing the type of eggplant fruit to cultivate. In addition, eggplants are found to contain high levels of antioxidant compounds, as well as phenolic and flavonoid compounds [4]. To explore the diversity of eggplant fruit metabolites, metabolomics—the comprehensive analysis of metabolites in a biological sample at a given time or condition—can be used [5]. Such metabolite profiling can be done using gas chromatography or liquid chromatography coupled with mass spectrometry (GC-MS or LC-MS). One approach, called untargeted metabolomics analysis, aims to collect as much information as possible from the metabolites contained in biological samples [6]. This approach has been successfully used to determine the metabolite profile of various plants such as tomato, arabidopsis, date, potato, and black cumin [7,8,9,10]. Information on the diverse metabolites of plants is very useful for better understanding of the genotypic or phenotypic differences of plants. In coffee, untargeted metabolomics analysis was used as a preliminary approach to determine the origin of coffee, after which targeted analysis was done to determine the difference in flavor and taste [11]. Furthermore, the usefulness of metabolomics in determining markers for discriminating original “kopi luwak” from adulterated coffee has also been reported [12]. In this research, we analyzed the diversity of metabolites found in different eggplant fruit accessions and their relationship with fruit morphology. This information will be beneficial for the development of strategies for improvement of the eggplant crop in the future. 

## 2. Results

### 2.1. Untargeted Metabolomics Analysis

**Figure 1:**
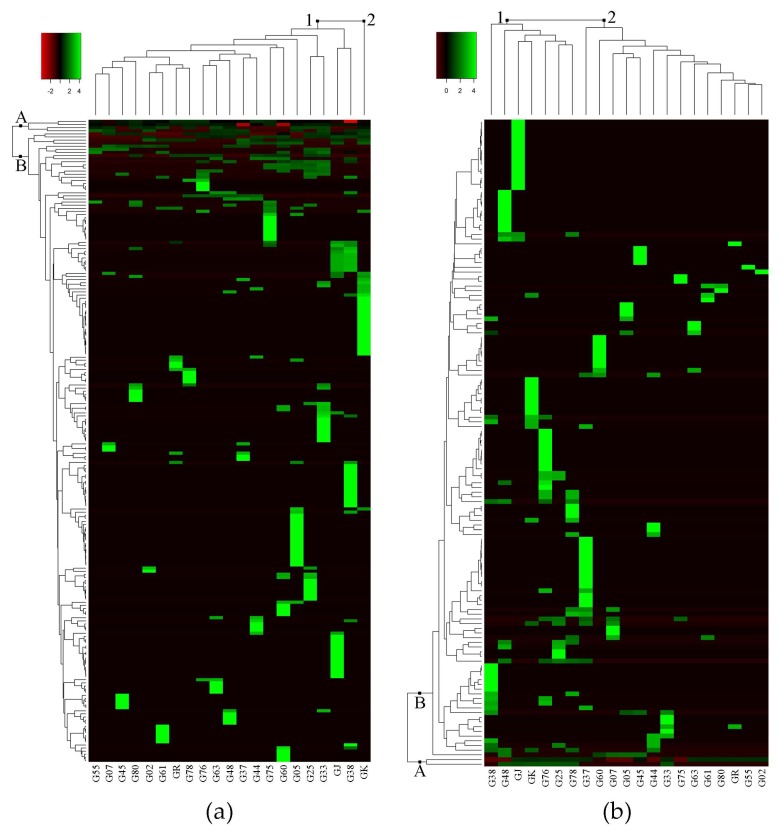
Heat map result of hierarchical group analysis based on metabolite peaks identified from LC-MS and GC-MS analyses. (**a**) heat map of 207 metabolite peaks from GC-MS analysis; (**b**) heat map of 136 metabolite peaks from LC-MS analysis. A = metabolites found in many eggplant accessions; B = metabolites found in a few, or only one, eggplant accession. The green, black, and red bars represent the value of area pct. that has been transformed by log2. The bright green color shows higher peak areas of metabolite, while black and red colors show lower peak areas of metabolites. This coloring is metabolite-wise.

Untargeted metabolomics analysis of eggplant fruit successfully detected 207 metabolite peaks using GC-MS, and 136 metabolite peaks using LC-MS. The log-transformed dataset of peak area percentage (area pct.) was used to construct the heat map and dendrogram (Figure 1). The heat map shows the metabolites that were detected in 21 eggplant accessions. Hierarchical cluster analyses (HCA), using Euclidean distance and complete linkage agglomerative methods, were carried out to measure the similarity of multivariate samples [13] and cluster the metabolomics data, respectively [14]. In the heat map, rows represent each detected metabolite, and columns represent the different eggplant accessions. Large peak areas were colored green, while smaller peak areas were black, and went red as peaks became smaller. Among the detected metabolite peaks from GC-MS and LC-MS analyses, 207 and 51 metabolites were putatively identified using the online metabolite databases MassBank (http://www.massbank.jp/?lang=en) and Plant Metabolic Pathway Databases (https://www.plantcyc.org/) [15,16] (Table 1 and Table 2), respectively. The important metabolites identified in the GC-MS analysis are shown in Table 1, and the complete list is shown in Table S1.

Figure 1a shows the heat map generated from GC-MS analysis. The detected metabolite peaks (in rows) produced two clusters: group A and group B, while eggplant accessions (in columns) also clustered into two groups: group 1 and group 2. Group A in the GC-MS heat map (Figure 1a) consisted of metabolites that were present in most of the eggplant accessions such as linoleic acid, palmitic acid, and neophytadiene (Table 1). Based on GC-MS analysis, the clustering of eggplant accessions showed interesting results in which only GK separated from the rest of the accessions. Group 1 consisted of 20 eggplant accessions while Group 2 consisted of only GK (Figure 1a). Accession-specific metabolites (indicated in green) for GK include linoleic acid, palmitic acid, and neophytadiene. Moreover, GK had the most accession-specific metabolites among all the samples (Table S1). On the other hand, accessions G37, G78 and GR each had a solitary accession-specific metabolite, namely solanesol, cyclododecanone, and (9E,12E)-9,12-octadecadienoyl chloride, respectively (Table 1).

Group A in the LC-MS heat map consisted of two unidentified metabolites, (LmUi 1) and (LmUi 2), which were present in 15 and 7 accessions, respectively. The other group of metabolites, group B, consisted of metabolites that were present in fewer eggplant accessions than group A. For instance, farnesyl acetone and citronellyl formate were found only in G02 and G25, respectively (Table 1). Likewise, L-dopachromate can be found in G37 and G38, cyclopentolate in G76 and G78, and carprofen in G37 (Table 2). 

The number of accession-specific metabolites was also the main factor for the clustering of groups in the LC-MS heat map (Figure 1b). As seen on the heat map, group 1, which includes G38, G48, GJ, GK, G76, G25, and G78, had more accession-specific metabolites (indicated in green) than group 2. The specific metabolites for each eggplant accession that have been annotated are shown in Table 2. Fifteen out of 21 accessions have annotated accession-specific metabolites, while six (G02, G25, G33, G44, G75, and G80) have unknowns. Table 2 shows 12 classes of identified metabolites, in which the alkaloid group was the most common metabolite class with 16 metabolites. There are four identified metabolites that can be found in more than two eggplant accessions, namely 2,5-bis(*N*-hexylmethylsilyl)thiophene, penicillin K, 2-(methylthiomethyl)-3-phenyl-2-propenal, and dimethisterone (Table 2). 

### 2.2. Correlation Analysis of Fruit Metabolites with Fruit Morphology

To gain insight on the relationship between fruit metabolites and morphology, data on the fruit morphology of 21 eggplant accessions were used in the correlation analysis. All eggplant accessions possess metabolites and fruit morphology characteristics that are unique to each accession (Table 3). Specifically, different eggplant accessions have different fruit size, shape, and color, with some also showing unique morphology such as fruit with patches and stripes on the skin (G02, G05, G48), fruit with ribs (G02, G05, G25 and G63), very dark purple fruit (GJ), and yellow fruit (GK).

**Figure 2:**
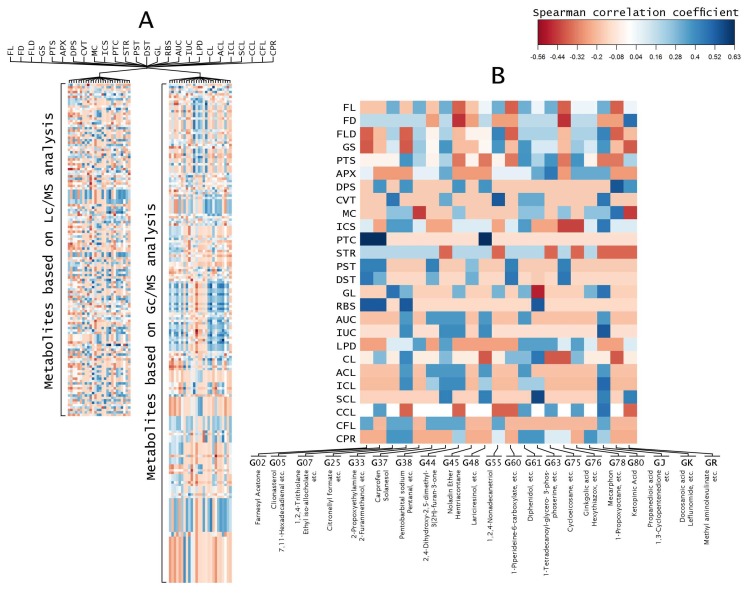
Heat map of correlations between eggplant fruit metabolites and fruit morphologies. Each square represents the Spearman’s correlation coefficient (*p* < 0.05). (**A**) Heat map of correlations between eggplant fruit metabolites based on 136 detected peaks of LC-MS analysis and 207 detected peaks of GC-MS analysis with 26 fruit morphologies; (**B**) Heat map of correlations between specific eggplant fruit metabolites of each accession with 26 fruit morphologies.

Correlation coefficient analysis indicated that fruit morphologies are related to metabolites (Figure 2). The correlation analysis was done between 26 fruit morphologies and all the identified peaks from the LC-MS and GC-MS analyses separately (Figure 2A). Furthermore, the correlation coefficient of accession-specific metabolites from each eggplant accession were collected and used to build a new heat map (Figure 2B and Table S2). Accession-specific metabolites from the same eggplant accessions showed similar coefficient correlation (Figure 2B). Out of 26 fruit morphology categories, seven eggplant fruit morphologies, namely DPS, CVT, PTC, DST, RBS, IUC, and SCL, showed correlations with specific metabolites. Accessions with patched fruit, G02, G05, and G48, correlated with farnesyl acetone, clionasterol, and lariciresinol, respectively. G25 along with G05 and G02, which have medium ribs, also showed a correlation between its fruit morphology and its accession-specific metabolites. Strong curvature fruit (CVT), which is unique to G55 fruit, was correlated with 1,2,4-nonadecanetriol. Similarly, very strong spininess of calyx (SCL) that is only found in G63, correlated with ethyl 9-heptadecenoate and 1-tetradecanoyl-glycero-3-phosphoserine metabolite, with the highest correlation coefficient. GJ with its very strong intensity of anthocyanin coloration underneath the calyx (IUC), and GK with its medium depth of indentation of pistil scar (DPS), also showed correlations with their metabolites. On the other hand, the rest of the accessions did not show any correlations.

## 3. Discussion

### 3.1. Untargeted Metabolomics Analysis Revealed Unique Metabolites Present in Different Accessions

Untargeted metabolomics analysis was successfully carried out using LC-MS and GC-MS on eggplant fruits. In this study, we used both GC-MS and LC-MS to increase the number of metabolites that could be detected due to the difference in the type of metabolites that can be analyzed by each analytical platform. GC-MS is more widely used in metabolomics analysis than LC-MS because there are more metabolite databases available for GC-MS [17]. However, using both LC-MS and GC-MS analysis is preferred in metabolomics. Choi et al. [11] used LC-MS and GC-MS in untargeted metabolomics analysis to determine the origin of coffee, followed by targeted analysis to determine the difference in flavor and taste. To assess the variety of our detected metabolites, data analysis was conducted using R software (Version 3.2.2) with the Metabolomics package. The heat map was able to show the differences in metabolites that were present in 21 eggplant accessions.

The metabolites identified in the different eggplant accessions were diverse, with some that are reported to have economic or health benefits. Using GC-MS analysis, we found 20 classes of metabolites, with terpenoids being the most abundant (Table 1). Farnesyl acetone, citronellyl formate, and 2-furanmethanol, in G02, G25, and G33 respectively, are terpenoids known as food additives [18,19,20]. We observed that most of the eggplant accessions have linoleic acid, palmitic acid, α-tocopherol (Vitamin E), and neophytadiene. Palmitic acid is a saturated fatty acid that is commonly found in plants and animals [21], while linoleic acid is one of the saturated fatty acids that is beneficial for acne-prone patients as a comedolytic agent [22] and for human bone health [23]. Moreover, these fatty acids are also important for the plant, as plant membranes contain a mixture of saturated and unsaturated fatty acids that are believed to be essential for the plant to adapt to environmental changes [24]. On the other hand, Vitamin E is commonly found in fruits and vegetables and is reported to have antioxidant activity [25]. Neophytadiene is a natural volatile compound found in tobacco, that contributes to its flavor in small effect and has antimicrobial activity [26].

Plants synthesize a wide variety of organic compounds. We also found a wide range of alkaloids, steroid, terpenes, fatty acids, and flavonoids in eggplants from the LC-MS analysis. These diverse organic compounds are believed to be part of an evolutionary process of plant defense against pests, diseases, droughts and other environmental challenges [27]. Other metabolites that were found in more than four accessions were 2,5-Bis(*N*-hexylmethylsilyl)thiophene and 2-(methylthiomethyl)-3-phenyl-2-propenal (Table 1). 2,5-Bis(*N*-hexylmethylsilyl)thiophene, which was identified using the metabolite database of Massbank of Japan Science and Technology Agency, has unknown function while 2-(methylthiomethyl)-3-phenyl-2-propenal is known as a food additive agent according to the JECFA (Joint FAO/WHO Expert Committee on Food Additives) database [28]. Some metabolites from the LC-MS analysis are still unidentified.

Analytical instruments used in this analysis were coupled with a low-resolution mass spectrometer. Low-resolution instruments are more cost effective and easier to access in newly industrialized countries such as Indonesia; thus, these instruments are suitable for use in the initial screening of local eggplant fruit metabolites. The drawbacks of using low-resolution LC/MS include poor mass accuracy and low specificity, thus it tends to yield several candidate metabolites for a particular peak. Therefore, metabolite annotation was performed by spectral library matching as a first step. Several candidate metabolites were then shortlisted and metabolites that showed identical names from both the MassBank and PlantCyc online databases were selected. Afterward, we double- checked in the public repository websites such as PubChem (https://pubchem.ncbi.nlm.nih.gov/) and ChemSpider (http://www.chemspider.com/) [29,30] to determine whether these metabolites are commonly found in plants.

### 3.2. Several Metabolites Are Accession-Specific

We described accession-specific metabolites as those that were present in only one accession (Table 1 and Table 2). For instance, mecarphon, (Table 2) which was present only in G78, is a natural pesticide that is used in seed treatment of *Delia* spp. [31]. Solanesol, which was present only in G37, is a metabolite that is mainly found in solanaceous crops, including tobacco, tomato, potato, eggplant, and pepper. Solanesol is used as a critical intermediate for the synthesis of ubiquinone drugs in the pharmaceutical industry [32]. Information on solanesol found in G37 might be used for the pharmaceutical industry as a useful backup source of solanesol, other than tobacco leaves.

The detection of specific metabolites in different eggplant accessions is likely dependent on the genetic makeup of the plant. Untargeted metabolomics and quantitative trait locus (QTL) analysis of *Arabidopsis thaliana* showed that metabolite variation is genetically controlled [33]. The long process of gene expression involves many metabolic pathways until it can finally manifest as a phenotype. Cellular regulatory processes produce metabolites as their end products to respond to biological signals and environmental changes that will affect the phenotype [34]. Similar to eggplant, metabolomics analysis of tomatoes and potatoes also showed differences in metabolites based on accessions [35,36]. Since analysis on the metabolomics level is closer to the expression of the plant phenotype than to the genomic level, it can better explain the phenotype. Therefore, metabolomics analysis is an appropriate approach to distinguish phenotypes [37]. This information on accession-specific metabolites can be used by plant breeders to choose and develop better eggplant varieties, as well as being a new selling point to consumers.

### 3.3. Correlation Analysis Shows Relationship between Fruit Morphologies and Metabolites

Creating variation in eggplant fruit size and shape is one of the objectives of breeding. Size and shape, along with taste, are the three principal qualities considered in the process of domestication of eggplant in China [38]. Selection and breeding over hundreds of years have resulted in a large number of eggplant varieties [39]. Genetic studies on fruit size and shape development in eggplant showed that these characteristics are controlled by some of the loci mapped to the common region of the genome [40]. Our eggplant accessions showed the variety in fruit color, including green, white, and purple, with only one accession with yellow fruit. Yellow eggplant is commonly used as an ornamental plant and not for food [41]. Purple, green, striped and patched eggplants are preferred in Southeast Asia over yellow eggplants [42]. This is probably the reason yellow eggplants are less common than others.

This research was able to obtain information about the distinct metabolites in different eggplant accessions and their correlations with fruit characteristics. Metabolites in the G33, G37, G38, G61, G78, and G80 accessions did not correlate with any fruit characteristics. We suspect that metabolites in these accessions are not included in the regulatory processes that lead to the phenotype of fruits. Recent studies about plant tissue-specific metabolism showed that some tissues with specific metabolite groups have interdependencies among them in terms of metabolite content [43]. Since metabolites are the result of interactions between the genome system and the environment that is not only the end result of gene expression but part of the regulation of the biological system [44], this might explain why some metabolites are not related to some characteristics.

Plant metabolomics is a continuously expanding field and, so far, recent studies are more focused on the application of metabolomics with other “omics” such as genomics, transcriptomics, and proteomics [43]. However, the vast number of metabolites in plants and their biological systems are still a challenge for metabolomics research. Metabolic alterations in lettuce (*Lactuca sativa* L.) caused by different CECs (contaminants of emerging concern) from exposure to irrigation water were linked to the changes in morphological characteristics such as leaf height and stem width [45]. This indicates that plant metabolites are correlated with plant morphological characteristics. However, the change in plant metabolites due to a sudden change of environment usually affects quantitative traits. On the other hand, our findings showed correlations between plant metabolites and morphological characteristics of eggplant for both quantitative (fruit length, fruit diameter) and qualitative traits (fruit main color, fruit patches). This study will be useful to further studies for developing preferred fruit morphology based on the metabolites contained therein.

Metabolomics analysis is an appropriate approach to distinguish phenotypes [37]. Information on metabolites that are correlated with morphological characteristics would be very useful for plant breeding strategies in eggplant. Moreover, plant breeders and consumers can use this information to make a quick assessment of eggplant fruits and their content. 

## 4. Materials and Methods

### 4.1. Plant Materials

A total of 21 eggplant fruit accessions with different fruit morphologies from the Center for Tropical Horticulture Studies, Bogor Agricultural University, Indonesia were used in this study (Table 4). The plants were maintained in Bogor, West Java province, Indonesia (altitude 265 m above sea level, maximum temperature 29 °C, minimum temperature 20 °C). Eggplant seeds were sowed and watered regularly for a five-week period in a greenhouse. The seedlings were transplanted manually into the experimental plot. Ten plants per accession were planted with three replications. Plants were spaced 30 cm between rows and 40 cm between columns. A combination of compound fertilizer and manure was used to ensure that plants grew normally. All plants were kept free from pest and disease using a combination of manual and chemical controls. 

### 4.2. Extraction and Sample Preparation

Eggplant fruits were harvested 12–15 days after anthesis [4]. Three eggplant fruits were sampled from each accession. The freshly-picked fruits were immediately cut into smaller sizes and dried using an oven at 40 °C for three days [46]. The small pieces from three eggplant fruits of each accession were pooled to make a representative fruit sample. Later, the dried fruit samples were placed in 99.9% pure ethanol and macerated for three days. Thirty microliters (30 µL) of the macerated samples were injected into the LC-MS instrument for analysis. For GC-MS analysis, 10 mL of the macerated samples were transferred to a new tube and evaporated for 1 h at 40 °C. The evaporated sample was added to 200 µL extract of the macerated samples to increase the concentration. Five microliters of sample was used for injection to get a wide range spectrum.

### 4.3. LC-MS Analysis

Waters Alliance 2695 HPLC system was used in the LC-MS analysis using an XTerra MS C18 column (2.1 × 100 mm, 3.5 µm particle size; Waters, MA, USA). The injection volume was 30 µL. The mobile phase A was methanol, while the mobile phase B was water with the following linear gradient programme: the concentration of mobile phase A was 10% at 0 min, 25% at 5 min, increased to 75% with gradient 5%/min and held for 5 min, then decreased to 10% and held for 15 min, while the mobile phase B was 90% at 0 min, 75% at 5 min, decreased to 25% and held for 5 min with gradient 5%/min, then increased until 90% and held for 15 min. The flow rate was 0.2 mL/min and the column temperature was 40 °C. The spectra were monitored using Waters Quattro Micro™ in full scan mode (*m*/*z* 50–1200) and electrospray ionization (ESI) interface in positive mode, with the source temperature maintained at 120 °C, desolvation temperature at 450 °C, and gas flow of 500 L/h. This procedure was controlled by Masslynx software (Version 4.0).

### 4.4. GC-MS Analysis

An Agilent Technologies 7890 A Gas Chromatograph coupled with 5975 C Mass Spectrometer system was used for analysis, with HP Ultra 2 capillary column (30 m, 0.25 mm i.d., 0.25 mm film thickness; Agilent, Santa Clara, CA, USA). The injection volume was 5 µL with 8:1 split ratio and 250 °C injection port temperature. The initial oven temperature was at 70 °C held for 0 min, increased at 3 °C/min to 150 °C, held for 1 min, and finally raised 20 °C/min to 250 °C and held for 26 min. Helium was used as carrier gas with a constant flow rate of 1.2 mL/min. MS acquisition parameters were done at 70 eV electron impact ionization, EM voltage of 2318 V, source 230 °C, quadrupole 150 °C, solvent delay: 2.5 min, and full scan (40–650 a.m.u) at a scan rate of 2.42 scan s^−1^. 

### 4.5. Putative Identification of Metabolites

Putative identification of the resulting data of LC-MS analysis was carried out using MZmine2 (Version 2.24) software [47]. The following parameters were used: (1) filter: Savitzky-Golay, number of datapoints: 5; (2) mass detector: centroid, MS level: 1, noise level: 1000; (3) chromatogram builder, min time span: 0.08 min, min height: 0.0E0, *m*/*z* tolerance: 0.001 *m*/*z* or 5 ppm; (4) peak detection using online database search option, Massbank of Japan Science and Technology Agency [15], Plant Metabolic Network: Plant Metabolic Pathway Databases [16]. For one detected peak we got a variety of metabolite candidates from a 1–10 name. We picked metabolites that showed the same name from both MassBank and PlantCyc online databases and commonly found in plants.

The data acquisition of GC-MS analysis was done using MS-Chemstation G1701-DA with WILEY and NIST spectral libraries. Volatiles that showed mass spectra with match factors quality of ≥90% were considered as putatively identified substances. The putatively identified peak list from both LC-MS and GC-MS, which showed the data set of retention time, peak height, and area pct. (% of the total measured area in the total ion chromatogram) was exported and processed in Microsoft Excel.

### 4.6. Fruit Morphology Evaluation

Eggplant fruits from 21 eggplant accessions were evaluated using characteristics in the Guidelines for the Conduct of Tests for Distinctness, Uniformity, and Stability (GCT-DUS) for Eggplant species [48]. These guidelines include 12 vegetative characteristics, 3 inflorescence characteristics, 26 fruit characteristics and 2 characteristics on the time of flowering and ripeness. For this research, we observed 26 fruit characteristics (Table 3). Three biological replicates from 10 fruits of each accession were used in evaluation of fruit morphology. The data were used for correlation analysis.

### 4.7. Data Analysis

The dataset of area pct. of metabolites was log-transformed and then analyzed using R software [49]. The Metabolomics package [50] for R software was used to perform HCA (hierarchical cluster analysis) and construct heat maps with dendrograms. HCA was performed using the Euclidean distance method and the complete linkage agglomerative method. 

The observed data from fruit morphology observation and the dataset of metabolites of eggplant accessions were used for correlation analysis. The Spearman correlation coefficients was calculated between these data. Calculations were done one by one between fruit morphology and its metabolites. Correlation analysis was conducted to determine the relation between eggplant fruit morphology and its metabolites. All calculations were done using the R software with the Corrplot package [51].

## 5. Conclusions

Untargeted metabolomics analysis was able to determine the metabolites contained in the eggplant fruit. We were able to detect 136 and 207 metabolite peaks from LC-MS and GC-MS analyses, respectively. Some of the metabolites were annotated as alkaloids, terpenes, terpenoids, fatty acids, and flavonoids. This study also found specific metabolites that are unique to a specific eggplant accession. Furthermore, Spearman correlation analysis showed the relationship between specific metabolites and fruit morphologies. Our results indicated that some specific metabolites in particular eggplant accessions correlated with its fruit morphologies. We believe that this information would be valuable for variety improvement program of eggplant.

## Figures and Tables

**Table 1:** Metabolite compounds from untargeted metabolomics analysis in eggplant fruit using GC-MS.

Name	Formula	Class	Accessions
Linoleic acid	C_18_H_32_O_2_	Saturated Fatty Acid	G02, G05, G07, G25, G33, G37, G44, G45, G48, G55, G60, G61, G63, G75, G76, G78, G80, GJ, GK, GR
Palmitic acid	C_16_H_32_O_2_	Saturated Fatty Acid	G02, G05, G07, G25, G33, G38, G44, G45, G48, G55, G61, G63, G75, G76, G78, G80, GJ, GK, GR
α-Tocopherol (Vitamin E)	C_29_H_50_O_2_	Vitamin	G07, G25, G33, G37, G38, G45, G48, G60, G63, G76, G78, G80, GJ, GK
Neophytadiene	C_20_H_38_	Diterpenoid	G02, G05, G07, G33, G37, G38, G48, G55, G63, GK
Farnesyl acetone	C_18_H_30_O	Terpenoid	G02
*N*,*N*-Dimethylethylamine	C_14_H_11_N	Amines	G05
Hexanal	C_6_H_12_O	Alkaloid	G05
2-Hydroxyethylphosphine	C_2_H_7_OP	Flavonoid	G05
(Dimethylamino)acetone	C_5_H_11_NO	Alkaloid	G05
4-Oxononanedioic acid	C_9_H_14_O_5_	Terpen	G05
Clionasterol	C_29_H_50_O	Triterpenoid	G05
7,11-Hexadecadienal	C_16_H_28_O	Pheromon	G05
Ethyl iso-allocholate	C_26_H_44_O_5_	Steroid	G07
1,1-Dibutylhydrazine	C_8_H_20_N_2_	Skeletal formula	G25
Citronellyl formate	C_11_H_20_O	Terpenoid	G25
13-Tetradecenal	C_14_H_26_O	Terpenoid	G25
Ammonium oxalate, monohydrate	C_2_H_8_N_2_O_4_	Terpenoid	G33
2-Propoxyethylamine	C_5_H_13_NO	Terpenoid	G33
2-Furanmethanol	C_5_H_6_O_2_	Terpenoid	G33
4-Methyl-1,3-dioxane	C_5_H_10_O_2_	Benzene and Subtituent Derivatives	G33
Ethyl tetradecanoate	C_16_H_32_O_2_	Unsaturated Fatty Acid	G33
Methyl (9E,12Z)-9,12-octadecadienoate	C_19_H_34_O_2_	Diterpenoid	G37
Solanesol	C_45_H_74_O	Polyterpen	G37
Butanoic acid, 3-hydroxy-	C_4_H_8_O_3_	Hydroxy Acid and Derivatives	G38
Methoxyethylamine	C_3_H_9_NO	Alkaloid	G38
1,4-Cyclohexanediol, trans-	C_6_H_12_O_2_	Flavonoid	G38
3-Butenoic acid	C_4_H_6_O_2_	Fatty Acids and Conjugate	G38
Pentanal	C_5_H_10_O	Saturated Fatty Acid	G38
E-9-Tetradecenal	C_14_H_26_O	Unsaturated Fatty Acid	G38
Methyl palmitoleate	C_17_H_32_O_2_	Fatty Acid Methyl Ester	G38
Octadecyltrichlorosilane	C_18_H_37_Cl_3_Si	Organochlorosilane	G38
2,4-Dihydroxy-2,5-dimethyl-3(2H)-furan-3-one	C_6_H_8_O_4_	Flavonoid (Ketone bodies)	G44
Hentriacontane	C_31_H_64_	Alkanes	G45
Diisooctyl phthalate	C_24_H_38_O_4_	Diterpenoid	G48
9,10-Dibromopentacosane	C_25_H_50_Br_2_	Recolcinolic lipid	G48
Hydroperoxide, 1-methylhexyl	C_7_H_16_O_2_	Terpenoid	G60
Eicosanoic acid	C_20_H_40_O_2_	Saturated Fatty Acid	G60
Octadecanoic acid	C_18_H_36_O_2_	Saturated Fatty Acid	G60
Ethyl 9-hexadecenoate	C_18_H_34_O_2_	Unsaturated Fatty Acid	G60
2,2-Dideuteropropane	C_3_H_8_	Hydrocarbon	G61
Tetradecanal	C_14_H_28_O	Saturated Fatty Acid	G61
Ethyl 9-heptadecenoate	C_19_H_38_O_2_	Saturated Fatty Acid	G63
Propanamide, N,N-dimethyl-	C_5_H_9_NO_2_	Steroid	G75
Cycloeicosane	C_20_H_40_	Terpen	G75
trans-Chrysanthemal	C_10_H_16_O	Natural pesticide	G75
Z,E-3,13-Octadecadien-1-ol	C_18_H_34_O	Unsaturated Fatty Acid	G76
1-Propoxyoctane	C_11_H_24_O	Steroid	G78
Cyclododecanone	C_12_H_22_O	Flavonoid (Ketone bodies)	G78
Ketopinic Acid	C_10_H_14_O_3_	Flavonoid	G80
Propanedioic acid	C_3_H_4_O_4_	Terpenoid	GJ
3-Amino-2-oxazolidinone	C_3_H_6_N_2_O_2_	Alkaloid	GJ
1,3-Cyclopentenedione	C_5_H_6_O_2_	Alkaloid	GJ
Trichloroacetic acid, undec-10-enyl ester	C_13_H_21_Cl_3_O_2_	Trichloroacetic Acid	GJ
Cyclohexadecanone	C_16_H_30_O	Organohalogen compound	GJ
Oleyl alcohol, heptafluorobutyrate	C_22_H_35_F_7_O_2_	Alkanes	GJ
Ethyl octadecanoate	C_20_H_40_O_2_	Unsaturated Fatty Acid	GK
10-HeptyL-10-Octylicosane	C_35_H_72_	Terpen	GK
Pentacosane	C_25_H_52_	Alkanes	GK
1-Chloroheptacosane	C_27_H_55_Cl	Alkanes	GK
Triacontane	C_30_H_62_	Alkanes	GK
9-Hexacosene	C_26_H_52_	Terpen	GK
Nonadecane	C_19_H_40_	Terpen	GK
(9E,12E)-9,12-Octadecadienoyl chloride	C_18_H_31_ClO	Skeletal formula	GR

**Table 2:** Metabolites from untargeted metabolomics analysis in eggplant fruit using LC-MS.

Name	Formula	Class	Accessions
2,5-Bis(*N*-hexylmethylsilyl)thiophene	C_18_H_36_SSi_2_	Terpen	G07, G25, G37, G76, G78
2-(Methylthiomethyl)-3-phenyl-2-propenal	C_11_H_12_OS	Terpenoid	G05, G33, G38, G45
Boscalid	C_18_H_12_C_l2_N_2_O	Alkaloids	G07, G25, G37, GK
Dimethisterone	C_23_H_32_O_2_	Steroid	G25, G48, G78
Glucolepidiin	C_17_H_14_Cl_2_N_2_O_2_	Alkaloids	G48, G78, GJ
Methyl-2-alpha-l-fucopyranosyl-beta-d-galactoside	C_13_H_24_O_10_	Glycoside	G25, G48
L-dopachromate	C_9_H_6_NO_4_	Terpenoid	G37, G38
L-saccharopine	C_11_H_19_N_2_O_6_	Alpha amino acids	G37, GK
Coumachlor	C_19_H_15_ClO_4_	Steroid	G37, G78
5-Methoxytryptamine	C_11_H_14_N_2_O	Alkaloids	G38, G78
Clopidol	C_7_H_7_Cl_2_NO	Alkaloids	G44, G60
4,4′-Ditolylthiourea	C_15_H_16_N_2_S	Steroid	G48, GJ
Octylbenzene	C_14_H_22_	Terpenoid	G76, G78
Cyclopentolate	C_17_H_25_NO_3_	Steroid	G76, G78
Trioxilin A3	C_20_H_33_O_5_	Steroid	G78, GK
Pyrazinemethanethiol	C_5_H_6_N_2_S	Monoterpene	G05
1-Diethoxyphosphoryl-4-hydroxy-nonan-2-one	C_13_H_27_O_5_P	Terpenoid	G05
1,2,4-Trithiolane	C_2_H_4_S_3_	Steroid	G07
2-Chloro-1,4-naphthoquinone	C_10_H_5_ClO_2_	Monoterpene	G37
Methyl 6-O-galloyl-beta-d-glucopyranoside	C_14_H_18_O_10_	Flavonoid	G37
Propericiazine	C_21_H_23_N_3_OS	Alkaloids	G37
N-Phenylacetylglutamic acid	C_13_H_17_NO_5_	Steroid	G37
Carprofen	C_15_H_12_ClNO_2_	Steroid	G37
8-O-Methyloblongine	C_20_H_26_NO_3_	Steroid	G37
5,5′-Methylenedi(2-para-tolylperhydropyrrolo(3,4-c)pyrrole-1,3-dione)	C_27_H_28_N4O_4_	Alkaloids	G38
1-Deoxy-d-xylulose	C_5_H_10_O_4_	Flavonoid	G38
Cis-Zeatin	C_10_H_13_N_5_O	Alkaloids	G38
1-Hydroperoxy-8-carboxyoctyl-3,4-epoxynon-(2E)-enyl-ether	C_12_H_18_BNO_2_	Alkaloids	G38
Pentobarbital sodium	C_11_H_17_N_2_O_3_	Alkaloids	G38
Noladin Ether	C_23_H_40_O_3_	Steroid	G45
Lariciresinol	C_20_H_24_O_6_	Monoterpene	G48
1,2-Bis(4-nitrophenyl)ethane	C_14_H_12_N_2_O_4_	Stilbenoid	G48
1,2,4-Nonadecanetriol	C_19_H_40_O_3_	Terpen	G55
1-Piperideine-6-carboxylate	C_6_H_8_NO_2_	Alkaloids	G60
9-Chloro-10-hydroxy-octadecanoic acid	C_18_H_35_ClO_3_	Alkaloids	G60
Diphenidol	C_21_H_27_NO	Monoterpene	G61
1-Tetradecanoyl-glycero-3-phosphoserine	C_20_H_40_NO_9_P	Fatty acid	G63
Ginkgolic acid	C_22_H_34_O_3_	Terpenoid	G76
Hexythiazox	C_17_H_21_ClN_2_O_2_S	Alkaloids	G76
Atovaquone	C_22_H_19_ClO_3_	Steroid	G76
5-Formiminotetrahydrofolate	C_20_H_24_N_8_O_6_	Monoterpene	G76
Butocarboxim	C_7_H_14_N_2_O_2_S	Alkaloids	G76
Elaeocarpidine	C_17_H_21_N_3_	Alkaloids	G76
Mecarphon	C_7_H_14_NO_4_PS_2_	Organophosphorus	G78
Ethyl 18-bromooctadec-17-en-5,7,15-triynoate	C_20_H_25_BrO_2_	Monoterpene	GJ
2-Acetoxy-7-bromo-4-isopropyltropone	C_12_H_13_BrO_3_	Terpen	GJ
*N*-(1-Deoxy-1-fructosyl)histidine	C_12_H_19_N_3_O_7_	Steroid	GJ
Docosanoic acid	C_22_H_44_O_2_	Terpen	GK
Leflunomide	C_12_H_9_F_3_N_2_O_2_	Steroid	GK
Yohimbinic acid	C_20_H_24_N_2_O_3_	Alkaloids	GK
Methyl aminolevulinate	C_6_H_11_NO_3_	Alkaloids	GR

**Table 3:** Description of fruit morphologies for 21 eggplant accessions based on UPOV.

Code	Morphological Characteristics																				
FL	Fruit: length (1) very short (<1 cm), (3) short (~ 2cm), (5) medium (~5 cm), (7) long (~10 cm), (9) very long (>20 cm)																				
FD	Fruit: maximum diameter (1) very small (<1 cm), (3) small (~2 cm), (5) medium (~3 cm), (7) large (~5 cm), (9) very large (>10 cm)																				
FLD	Fruit: ratio length/maximum diameter (1) very small, (3) small, (5) medium, (7) large, (9) very large																				
GS	Fruit: general shape (1) globular, (2) ovoid, (3) obovate, (4) pear shaped, (5) club shaped, (6) ellipsoid, (7) cylindrical																				
PTS	Fruit: size of pistil scar (1) very small, (3) small, (5) medium, (7) large, (9) very large																				
APX	Fruit: apex (1) indented, (2) flattened, (3) rounded, (4) pointed																				
DPS	Fruit: depth of indentation of pistil scar (1) absent or very shallow, (3) shallow, (5) medium, (7) deep, (9) very deep																				
CVT	Only for cylindrical types: Fruit: curvature (1) absent or very weak, (3) weak, (5) medium, (7) strong, (9) very strong																				
MC	Fruit: main color of skin at harvest maturity (1) white, (2) green, (3) violet, (4) yellow																				
ICS	Fruit: intensity of main color of skin (1) very light, (3) light, (5) medium, (7) dark, (9) very dark																				
PTC	Fruit: patches (1) absent, (9) present																				
STR	Fruit: stripes (1) absent, (9) present																				
PST	Fruit: prominence of stripes (3) weak, (5) medium, (7) strong																				
DST	Fruit: density of stripes (3) sparse, (5) medium, (7) dense																				
GL	Fruit: glossiness at harvest maturity (3) weak, (5) medium, (7) strong																				
RBS	Fruit: ribs (1) absent or very weak, (3) weak, (5) medium, (7) strong, (9) very strong																				
AUC	Fruit: anthocyanin coloration underneath calyx (1) absent, (9) present																				
IUC	Fruit: intensity of anthocyanin coloration underneath calyx (3) weak, (5) medium, (7) strong																				
LPD	Fruit: length of peduncle (1) very short, (3) short, (5) medium, (7) long, (9) very long																				
CL	Fruit: size of calyx (1) very small, (3) small, (5) medium, (7) large, (9) very large																				
ACL	Fruit: anthocyanin coloration of calyx (1) absent, (9) present																				
ICL	Fruit: intensity of anthocyanin coloration of calyx - (1) very weak, (3) weak, (5) medium, (7) strong, (9) very strong																				
SCL	Fruit: spininess of calyx (1) absent or very weak, (3) weak, (5) medium, (7) strong, (9) very strong																				
CCL	Fruit: creasing of calyx (1) very weak, (3) weak, (5) medium, (7) strong, (9) very strong																				
CFL	Fruit: color of flesh (1) whitish, (2) greenish																				
CPR	Fruit: color of skin at physiological ripeness (1) yellow, (2) orange, (3) ochre, (4) brown																				
**Code**	**G02**	**G05**	**G07**	**G25**	**G33**	**G37**	**G38**	**G44**	**G45**	**G48**	**G55**	**G60**	**G61**	**G63**	**G75**	**G76**	**G78**	**G80**	**GJ**	**GK**	**GR**
FL	5	5	9	5	9	5	9	3	5	7	9	3	9	7	9	3	7	7	9	3	7
FD	7	7	7	7	7	5	7	3	5	7	5	5	7	7	7	3	7	7	5	5	9
FLD	1	3	7	1	7	5	7	3	5	5	9	1	7	7	7	3	7	7	9	1	3
GS	1	2	5	1	6	3	7	2	2	3	7	2	7	5	5	2	6	5	7	2	1
PTS	5	5	5	9	7	5	7	3	5	3	5	3	9	7	9	3	9	5	3	3	7
APX	3	1	1	1	3	3	4	1	1	1	3	3	3	4	7	1	4	4	4	1	1
DPS	1	3	1	3	1	1	1	3	1	3	1	1	1	1	1	1	1	1	1	5	3
CVT	1	1	5	1	1	1	3	1	1	1	7	1	3	3	1	1	1	1	5	1	1
MC	2	2	3	3	1	2	2	3	2	2	2	2	3	3	2	3	2	3	3	5	1
ICS	5	3	7	7	5	3	3	5	5	5	3	5	7	3	3	1	1	5	9	5	5
PTC	9	9	1	1	1	1	1	1	1	9	1	1	1	1	1	1	1	1	1	1	1
STR	9	9	9	9	9	9	1	9	9	9	1	9	9	9	1	9	1	9	1	1	1
PST	7	7	3	3	3	7	3	3	5	3	3	7	3	3	3	7	3	3	3	3	3
DST	7	5	3	3	3	5	3	3	5	3	3	7	3	3	3	7	3	3	3	3	3
GL	3	3	7	5	3	3	3	5	3	3	5	3	5	1	3	3	3	5	7	3	3
RBS	5	5	1	5	1	1	1	1	1	1	1	1	1	5	1	1	1	1	1	1	1
AUC	1	1	1	9	1	9	9	9	1	9	1	1	9	1	1	1	1	1	9	1	1
IUC	3	3	3	5	3	3	5	5	3	5	3	3	3	3	3	3	3	3	7	3	3
LPD	5	5	7	7	9	5	9	5	5	5	5	5	9	9	9	7	9	7	5	5	7
CL	3	5	3	7	3	3	5	3	3	1	3	5	5	9	1	1	5	3	3	1	3
ACL	1	1	1	9	1	9	9	9	1	9	1	1	9	1	1	1	1	1	9	1	1
ICL	1	1	1	5	1	3	5	5	1	3	1	1	3	3	1	1	1	1	9	1	1
SCL	1	1	1	1	1	1	3	1	1	3	1	1	1	9	1	1	1	1	3	1	3
CCL	3	5	3	1	3	3	3	1	3	3	1	1	5	3	5	3	5	3	7	3	1
CFL	2	1	2	2	1	2	2	2	1	1	2	1	1	1	1	1	1	1	2	1	1
CPR	1	1	4	4	1	1	1	3	1	1	2	1	4	4	2	2	1	4	3	1	1

**Table 4:** Description of 21 eggplant accessions from the Center for Tropical Horticulture Studies.

Accessions Code	Fruit Morphology	
	Color	Shape
G02	Green with stripes and patches	Globular
G05	Light green with stripes and patches	Ovoid
G07	Dark purple with stripes	Club shaped
G25	Dark purple with stripes	Globular
G33	White with stripes	Ellipsoid
G37	Light green with stripes	Obovate
G38	Light green	Cylindrical
G44	Purple	Ovoid
G45	Green with stripes	Ovoid
G48	Green with stripes and patches	Obovate
G55	Light green	Cylindrical
G60	Green with stripes	Ovoid
G61	Dark purple	Cylindrical
G63	Light purple with stripes	Club shaped
G75	Light green	Club shaped
G76	Very light purple with green stripes	Ovoid
G78	Very light green	Ellipsoid
G80	Purple with stripes	Club shaped
GJ	Very dark purple	Cylindrical
GK	Yellow	Ovoid
GR	White	Globular

## Supplementary Materials

The following are available online at http://www.mdpi.com/2218-1989/8/3/49/s1, Table S1: Complete list of metabolites compounds from untargeted metabolomics analysis in eggplant fruit using GC-MS. Table S2: Spearman’s correlation coefficient value between eggplant fruit metabolites and fruit morphologies.
