# Supplementary material for: Untargeted Metabolomics Analysis of Eggplant (Solanum melongena L.) Fruit and Its Correlation to Fruit Morphologies
Source: Metabolites. 2018 Sep 1;8(3):49. doi: 10.3390/metabo8030049 (PMC6160926; doi:10.3390/metabo8030049)
Supplement: Supplementary file 1 [file metabolites-08-00049-s001.pdf]

metabolites

MDPI

Table S1. Complete list of metabolites compounds from untargeted metabolomics analysis in eggplant fruit using GC-MS

Name	Accessions
Linoleic acid	G02, G05, G07, G25, G33, G37, G44, G45, G48, G55, G60, G61, G63, G75, G76, G80, GJ, GK, GR
Palmitic acid	G02, G05,G07, G25, G33, G38, G44, G45, G48, G55, G61, G63, G75, G76,G78, G80,GJ, GK,GR
Vitamin E	G07, G25, G33, G37, G38, G45, G48, G60, G63, G76, G78, G80, GJ, GK
(Z,Z)-9,12-octadecadienoic acid	G02, G05, G07, G25, G33, G48, G55, G61, G63, G78, G80, GK
n-hexadecanoic acid	G02, G25, G33, G38, G44, G48, G60, G63, G75, G76, GJ, GK
Neophytadiene	G02, G05, G07, G33, G37, G38, G48, G55, G63, GK
Acetic acid	G25, G33, G44, G60, G63, G75, G76, G78, G80, GR
Spinacen	G02, G05, G37, G44, G60, G61, G78, GJ, GK, GR
Tricosane	G02, G07, G37, G45, G48, G60, G61, G80
2,3-dihydro-3,5-dihydroxy-6-methyl-4H-pyran-4-one	G05, G25, G33, G38, G48, G60, G76
Stigmastan-3,5-diene	G37, G38, G44, G48, G63, G76, G78
Hexadecanoic acid, ethyl ester	G05, G37, G44, G60, G75, GJ, GK
Octacosane	G25, G33, G38, G55, G60, G61
2-furancarboxaldehyde	G05, G25, G33, G60, G75
2-furancarboxaldehyde, 5-(hydroxymethyl)-	G05, G25, G33, G60, G75
(6e,10e,14e,18e)-2,6,10,15,19,23-hexamethyl-2,6,10,14,18,22-tetracosahexaene	G07, G25, G33, G45, G55
Formamide, n-methoxy-	G25, G33, G60, G63, G76
4h-pyran-4-one, 2,3-dihydro-3,5-dihydroxy-6-methyl-	G38, G44, G60, G63, G75
2h-1-benzopyran-6-ol, 3,4-dihydro-2,5,7,8-tetramethyl-2-(4,8,12-trimethyltridecyl)-, [2r-[2r*(4r*,8r*)]]-	G05, G61, G75, GR
Eicosanoic acid	G25, G33, G45, G76
Hexadecanoic acid, ethyl ester	G25, G38, G61, G76
Cholesta-4,6-dien-3-ol, 6-fluoro-, (3.beta.)-	G25, G48, G75, G80
Oleic acid	G38, G75, GJ, GR
Hexadecanoic acid, methyl ester	G05, G38, GR

Table S1. *Cont.*

Name	Accessions
(9z)-9,17-octadecadienal	G07, GJ, GK
Heptacosane	G33, G60, GK
1,2-15,16-diepoxyhexadecane	G33, G78, G80
Ethyl (9z,12z)-9,12-octadecadienoate	G37, G44, G48
Squalene	G38, G48, G63
2-propanol, 1-amino-	G38, G60
Cis-vaccenic acid	G38, G75, GJ
(9e)-9-octadecenoic acid	G38, G80, GJ
(6e,10e,14e,18e)-2,6,10,15,19,23-hexamethyl-2,6,10,14,18,22-tetracosahexaene	G55, G75, G80
Ascorbyl palmitate	G80, GJ, GK
Octadecanoic acid, methyl ester	G07, G37
4-methoxy-2-butyn-1-ol	G02, G25
3-d-4-methyl-2-pentanol	G02, GR
2-n-butyl-8-n-hexyldecahydronaphthalene	G05, G38
Benzoic acid, eicosyl ester	G05, G60
17-(1,5-dimethylhexyl)-2,3-dihydroxy-10,13-dimethyl-1,2,3,7,8,9,10,11,12,13,14,15,16,17-tetradecahydrocyclopenta[a]phenanthren-6-one	G05, G60
Hexacosanoic acid	G05, G75
Octadecanoic acid	G05, GK
Hydroxymethylfurfurole	G25, G33
1-butanol, 4-methoxy-	G25, G60
.Alpha.-dihydrofucosterol	G25, G60
2-methyl-z,z-3,13-octadecadienol	G33, G38
Tetradecanoic acid, ethyl ester	G33, G48
Oleyl alcohol	G33, G60
Tridecanedial	G33, G80
2,2-dimethyl-5-(3-methyl-2-oxiranyl)cyclohexanone	G33, GJ
11-decylheneicosane	G33, GK
Stigmast-5-en-3-ol	G33, GK
Octadecanoic acid, ethyl ester	G37, GK
Bicyclo[10.8.0]eicosane, cis-	G38, G44
Propanoic acid	G38, GJ
Ethylboronic acid	G38, GJ
D-(-)-3-acetylthioisobutyric acid	G38, GJ
N-acetyl-guanidine	G38, GJ
2(3h)-furanone, dihydro-	G38, GJ
Diltiazem	G38, GJ
Icosane	G38, GJ
Cycloartanol	G38, GK
S-methylpropanethiosulfonate	G44, G63
Pyrrolidine-.alpha.,.alpha.,.alpha.',.alpha.'-d4	G44, GJ
10-heptyl-10-octylicosane	G44, GK
2,3-butanediol	G44, GR

Table S1. *Cont.*

Name	Accessions
Z,z-10,12-hexadecadien-1-ol acetate	G48, GK
1,3-dioxolane, 4,5-dimethyl-, cis-	G63, G76
Ethyl cis,cis-9,12-octadecadienoate	G76, G78
Nonadecanoic acid	G78, GR
Farnesyl acetone	G02
Acetic acid	G05
N,n-dimethylethylamine	G05
Hexanal	G05
2-hydroxyethylphosphine	G05
5-(methoxycarbonyl)-1,1,3,3-tetramethyl-4-oxopiperidinium iodide	G05
Carbamic acid, 2-(dimethylamino)ethyl ester	G05
(Dimethylamino)acetone	G05
Ethanol, 2-methoxy-, acetate	G05
Nonanedioic acid, 4-oxo-	G05
11,12-dibromo-tetradecan-1-ol acetate	G05
9,12-octadecadien-1-ol	G05
Heptadecanoic acid, 15-methyl-, ethyl ester	G05
3-vinylcholestan-3-ol	G05
7,11-hexadecadienal	G05
Stigmasterol	G05
Clionasterol	G05
2-methyl-3-(3-methyl-but-2-enyl)-2-(4-methyl-pent-3-enyl)-oxetane	G07
Ethyl iso-allocholate	G07
1,1-dibutylhydrazine	G25
3-ethyl-1-thia-cyclopentane	G25
Citronellyl formate	G25
Benzenemethanamine, .alpha.,4-dimethyl-	G25
2-(4-methylcyclohexyl)-2-propen-1-ol	G25
13-tetradecenal	G25
Oxiraneundecanoic acid, 3-pentyl-, methyl ester, trans-	G25
Ammonium oxalate, monohydrate	G33
2-propoxyethanamine	G33
2-furanmethanol	G33
4-methyl-1,3-dioxane	G33
Talpinine	G33
1,2-benzenedicarboxylic acid, mono(2-ethylhexyl) ester	G33
Glycerol 1,3-dihexandecanoate-2-(.delta.-9, 12)-octadecadienoate	G33
10-butyl-10-propylicosane	G33
Solanesol	G37
2,3-dihydroxycholest-4-en-6-one	G37
Methyl (9e,12e)-9,12-octadecadienoate	G37
1,4-butane-1,1,4,4-d4-diamine	G38

Table S1. *Cont.*

Name	Accessions
Butanoic acid, 3-hydroxy-	G38
Methoxyethylamine	G38
S-allyl 3-oxobutanethioate	G38
1,4-cyclohexanediol, trans-	G38
1-deuteropropane	G38
3-butenoic acid	G38
Pentanal	G38
E-9-tetradecenal	G38
Methyl palmitoleate	G38
Methyl 17-methyl-octadecanoate	G38
Trans-13-octadecenoic acid	G38
2-phenanthrenol, 1,2,3,4,4a,4b,5,6,8a,9,10,10a-dodecahydro-4a,7-dimethyl-8-[3-cyano-3-(trimethylsilyloxy)propyl]-, acetate	G38
Silane, trichlorooctadecyl-	G38
6-nitro-cylohexadecane-1,3-dione	G38
2,4-dihydroxy-2,5-dimethyl-3(2h)-furan-3-one	G44
Bicyclo[10.8.0]eicosane, (e)-	G44
2,5,7,8-tetramethyl-2-(4,8,12-trimethyltridecyl)-6-chromanol	G44
(2e)-3,7,11,15-tetramethyl-2-hexadecen-1-ol	G45
Tetradecanoic acid	G45
9,12-octadecadienoic acid, ethyl ester	G45
Oxirane, 2,2-dimethyl-3-(3,7,12,16,20-pentamethyl-3,7,11,15,19-heneicosapentaenyl)-, (all-e)-	G45
Hentriacontane	G45
10-ethoxycarbonyl-9-oxa[3.3.2]propellane	G48
Diisooctyl phthalate	G48
3,4-seco-5.alpha.-cholestan-3-oic acid, 4-hydroxy-4-methyl-, .epsilon.-lactone, (4r)-	G48
9,10-dibromopentacosane	G48
Hydroperoxide, 1-methylhexyl	G60
1-(2-furyl)-2,3-dimethyl-1,2-butanediol	G61
2,2-dideuteropropane	G61
1,3-butanediol	G61
Tetradecanal	G61
(S)(+)-z-13-methyl-11-pentadecen-1-ol acetate	G61
2h-1,2,3-triazol-4-amine, 2-cyclohexyl-5-nitro-, 1-oxide	G61
.Beta.-sitosterol acetate	G61
Z-10-tetradecen-1-ol acetate	G62
Eicosanoic acid	G63
3-methoxy-2-butanol	G63
Ethyl 9-heptadecenoate	G63
3-mercapto-2-methyl-4,5-dihydrofuran	G63
2h-tetraazol-5-amine	G63
1,9-tetradecadiene	G64
Octadecanoic acid	G65

Table S1. *Cont.*

Name	Accessions
Ethyl 9-hexadecenoate	G66
Benzoic acid, octadecyl ester	G67
1-butanol, 4-methoxy-	G75
4-aminobutyraldehyde diethyl acetal	G75
Propanamide, n,n-dimethyl-	G75
2,6,6-trimethylbicyclo[3.1.1]heptane	G75
Cycloeicosane	G75
12-methyl-e,e-2,13-octadecadien-1-ol	G75
Tetracosanoic acid	G75
16-[(1-ethyl-3-methyl-1h-pyrazol-4-yl)methylene]androstane-3,17-diol	G75
Trans-chrysanthemal	G75
E,e-6,11-tridecadien-1-ol acetate	G76
Z,e-3,13-octadecadien-1-ol	G76
Cholestan-3,26-diol-22-oxime	G76
1-propoxyoctane	G78
Cyclododecanone	G78
1,3,4,5-tetrahydroxycyclohexanecarboxylic acid	G78
2-chloroethyl (9z,12z)-9,12-octadecadienoate	G78
2-propanol	G80
Ketopinic acid	G80
Methyl 14-methylpentadecanoate	G80
Linolsaeure	G80
Propanedioic acid	GJ
3-methyloxirane-2-carboxylic acid	GJ
1-amino-2-propanol	GJ
3-amino-2-oxazolidinone	GJ
1,3-cyclopentenedione	GJ
Trichloroacetic acid, undec-10-enyl ester	GJ
Palmitic acid, methyl ester	GJ
Trans-13-octadecenoic acid, methyl ester	GJ
Cyclohexadecanone	GJ
1-cinnamyl-3-methylindole-2-carbaldehyde	GJ
Linoleoyl chloride	GJ
1h-indazolobis(9-bora-bicyclo[3.3.1]nonyl)oxide	GJ
22,26-oxido-4,17-cholestadien-3.beta.,16.alpha.-diol	GJ
Oleyl alcohol, heptafluorobutyrate	GJ
(9e,12e)-9,12-octadecadienoyl chloride	GJ
Methyl 19-methyl-eicosanoate	GK
Pentacosane	GK
N7-methyladenine	GK
(8z)-14-methyl-8-hexadecen-1-ol	GK
1-chloroheptacosane	GK
Triacontane	GK
9-hexacosene	GK

Table S1. *Cont.*

Name	Accessions
Nonadecane	GK
.Gamma.-tocopheryl methyl ether	GK
17-(1,5-dimethylhexyl)-10,13-dimethyl-4-vinylhexadecahydrocyclopenta[a]phenanthren-3-ol	GK
Z,e-2,13-octadecadien-1-ol	GK
Dihydrolanosterin	GK
Citrost-7-en-3-ol	GK
2,7-dihydroxy-5-methoxy-3-methylanthraquinone ditms	GK
Cholestan-3-yl thiocyanate	GK
Silane, [[(3.beta.,22e)-ergosta-7,22-dien-3-yl]oxy]trimethyl-	GK
4-vinylcholestan-3-ol	GK
4,4-dimethylcholestan-3-one	GK
4-oxatricyclo[20.8.0.0(7,16)]triaconta-1(20),7(16)-diene	GR
Methyl 3-hydroxydodecanoate	GR

Table S2. Spearman's correlation coefficient value between eggplant fruit metabolites and fruit morphologies.

Code	G02	G05	G07	G25	G33	G37	G38	G44	G45	G48	G55	G60	G61	G63	G75	G76	G78	G80	GJ	GK	GR
FL	-0.15	-0.15	0.27	-0.15	0.27	-0.15	0.27	-0.33	-0.15	0.04	0.27	-0.33	0.27	0.04	0.27	-0.33	0.04	0.04	0.27	-0.33	0.04
FD	0.15	0.15	0.15	0.15	0.15	-0.23	0.15	-0.39	-0.23	0.15	-0.23	-0.23	0.15	0.15	0.15	-0.39	0.15	0.15	-0.23	-0.23	0.42
FLD	-0.33	-0.17	0.17	-0.33	0.17	-0.04	0.17	-0.17	-0.04	-0.04	0.36	-0.33	0.17	0.17	0.17	-0.17	0.17	0.17	0.36	-0.33	-0.17
GS	-0.34	-0.17	0.09	-0.34	0.21	-0.02	0.32	-0.17	-0.17	-0.02	0.32	-0.17	0.32	0.09	0.09	-0.17	0.21	0.09	0.32	-0.17	-0.34
PTS	-0.04	-0.04	-0.04	0.33	0.17	-0.04	0.17	-0.29	-0.04	-0.29	-0.04	-0.29	0.33	0.17	0.33	-0.29	0.33	-0.04	-0.29	-0.29	0.17
APX	0.06	-0.24	-0.24	-0.24	0.06	0.06	0.27	-0.24	-0.24	-0.24	0.06	0.06	0.06	0.27	0.39	-0.24	0.27	0.27	0.27	-0.24	-0.24
DPS	-0.14	0.33	-0.14	0.33	-0.14	-0.14	-0.14	0.33	-0.14	0.33	-0.14	-0.14	-0.14	-0.14	-0.14	-0.14	-0.14	-0.14	-0.14	0.47*	0.33
CVT	-0.14	-0.14	0.39	-0.14	-0.14	-0.14	0.28	-0.14	-0.14	-0.14	0.46*	-0.14	0.28	0.28	-0.14	-0.14	-0.14	-0.14	0.39	-0.14	-0.14
MC	-0.14	-0.14	0.22	0.22	-0.38	-0.14	-0.14	0.22	-0.14	-0.14	-0.14	-0.14	0.22	0.22	-0.14	0.22	-0.14	0.22	0.22	0.4	-0.38
ICS	0.08	-0.21	0.31	0.31	0.08	-0.21	-0.21	0.08	0.08	0.08	-0.21	0.08	0.31	-0.21	-0.21	-0.37	-0.37	0.08	0.39	0.08	0.08
PTC	0.55*	0.55*	-0.09	-0.09	-0.09	-0.09	-0.09	-0.09	-0.09	0.55*	-0.09	-0.09	-0.09	-0.09	-0.09	-0.09	-0.09	-0.09	-0.09	-0.09	-0.09
STR	0.16	0.16	0.16	0.16	0.16	0.16	-0.32	0.16	0.16	0.16	-0.32	0.16	0.16	0.16	-0.32	0.16	-0.32	0.16	-0.32	-0.32	-0.32
PST	0.37	0.37	-0.14	-0.14	-0.14	0.37	-0.14	-0.14	0.23	-0.14	-0.14	0.37	-0.14	-0.14	-0.14	0.37	-0.14	-0.14	-0.14	-0.14	-0.14
DST	0.4	0.26	-0.11	-0.11	-0.11	0.26	-0.11	-0.11	0.26	-0.11	-0.11	0.4	-0.11	-0.14	-0.11	0.4	-0.11	-0.11	-0.11	-0.11	-0.11
GL	-0.13	-0.13	0.41	0.26	-0.13	-0.13	-0.13	0.26	-0.13	-0.13	0.26	-0.13	0.26	-0.43	-0.13	-0.13	-0.13	0.26	0.41	-0.13	-0.13
RBS	0.46*	0.46*	-0.11	0.46*	-0.11	-0.11	-0.11	-0.11	-0.11	-0.11	-0.11	-0.11	-0.11	0.46*	-0.11	-0.11	-0.11	-0.11	-0.11	-0.11	-0.11
AUC	-0.16	-0.16	-0.16	0.32	-0.16	0.32	0.32	0.32	-0.16	0.32	-0.16	-0.16	0.32	-0.16	-0.16	-0.16	-0.16	-0.16	0.32	-0.16	-0.16
IUC	-0.09	-0.09	-0.09	0.35	-0.09	-0.09	0.35	0.35	-0.12	0.35	-0.09	-0.09	-0.09	-0.09	-0.09	-0.09	-0.09	-0.09	0.47*	-0.09	-0.09
LPD	-0.22	-0.22	0.08	0.08	0.3	-0.22	0.3	-0.22	-0.22	-0.22	-0.22	-0.22	0.3	0.3	0.3	0.08	0.3	0.08	-0.22	-0.22	0.08
CL	-0.06	0.24	-0.06	0.36	-0.06	-0.06	0.24	-0.06	-0.06	-0.34	-0.06	0.24	0.24	0.4	-0.34	-0.34	0.24	-0.06	-0.06	-0.34	-0.06
ACL	-0.16	-0.16	-0.16	0.32	-0.16	0.32	0.32	0.32	-0.16	0.32	-0.16	-0.16	0.32	-0.16	-0.16	-0.16	-0.16	-0.16	0.32	-0.16	-0.16
ICL	-0.17	-0.17	-0.17	0.34	-0.17	0.19	0.34	0.34	-0.17	0.19	-0.17	-0.17	0.19	0.19	-0.17	-0.17	-0.17	-0.17	0.43	-0.17	-0.17
SCL	-0.12	-0.12	-0.12	-0.12	-0.12	-0.12	0.37	-0.12	-0.12	0.37	-0.12	-0.12	-0.12	0.5*	-0.12	-0.12	-0.12	-0.12	0.37	-0.12	0.37
CCL	0	0.3	0	-0.32	0	0	0	-0.32	0	0	-0.32	-0.32	0.3	0	0.3	0	0.3	0	0.4	0	-0.32
CFL	0.29	-0.18	0.29	0.29	-0.18	0.29	0.29	0.29	-0.18	-0.18	0.29	-0.18	-0.18	-0.18	-0.18	-0.18	-0.18	-0.18	0.29	-0.18	-0.18
CPR	-0.2	-0.2	0.32	0.32	-0.2	-0.2	-0.2	0.18	-0.2	-0.2	0.08	-0.2	0.32	0.32	0.08	0.08	-0.2	0.32	0.18	-0.2	-0.2

* indicate significance at $P<0.05$
